# Long-Term Oral Administration of LLHK, LHK, and HK Alters Gene Expression Profile and Restores Age-Dependent Atrophy and Dysfunction of Rat Salivary Glands

**DOI:** 10.3390/biomedicines8020038

**Published:** 2020-02-20

**Authors:** Yasuko Ishikawa, Tomasz D Pieczonka, Aneta M Bragiel-Pieczonka, Harumichi Seta, Tadahiro Ohkuri, Yumi Sasanuma, Yuji Nonaka

**Affiliations:** 1 Department of Medical Pharmacology, Institute of Biomedical Sciences, Tokushima University Graduate School, 3-18-15, Kuramoto-cho, Tokushima 770-8504, Japan; td.pieczonka@gmail.com (T.D.P.); aneta.bragiel@gmail.com (A.M.B.-P.); 2 Suntory Global Innovation Center Ltd., Suntory World Research Center, 8-1-1 Seika-cho, Soraku-gun, Kyoto 619-0284, Japan; Harumichi_Seta@suntory.co.jp (H.S.); Tadahiro_Ohkuri@suntory.co.jp (T.O.); Yumi_Ando@suntory.co.jp (Y.S.); Yuji_Nonaka@suntory.co.jp (Y.N.)

**Keywords:** peptides, LHK, LHK, HK, Aging, DNA microarray, salivary glands, atrophy, dysfunction

## Abstract

Xerostomia, also known as dry mouth, is caused by a reduction in salivary secretion and by changes in the composition of saliva associated with the malfunction of salivary glands. Xerostomia decreases quality of life. In the present study, we investigated the effects of peptides derived from β-lactoglobulin C on age-dependent atrophy, gene expression profiles, and the dysfunction of salivary glands. Long-term oral administration of Leu^57^-Leu^58^-His^59^-Lys^60^ (LLHK), Leu^58^-His^59^-Lys^60^ (LHK) and His^59^-Lys^60^ (HK) peptides induced salivary secretion and prevented and/or reversed the age-dependent atrophy of salivary glands in older rats. The transcripts of 78 genes were upregulated and those of 81 genes were downregulated by more than 2.0-fold (*p ≤* 0.05) after LHK treatment. LHK upregulated major salivary protein genes such as proline-rich proteins (*Prpmp5*, *Prb3*, *Prp2, Prb1, Prp15*), cystatins (*Cst5*, *Cyss, Vegp2*), amylases (*Amy1a, Amy2a3*), and lysozyme (*Lyzl1*), suggesting that LLHK, LHK, and HK restored normal salivary function. The AP-2 transcription factor gene (*Tcfap2b*) was also induced significantly by LHK treatment. These results suggest that LLHK, LHK, and HK-administration may prevent and/or reverse the age-dependent atrophy and functional decline of salivary glands by affecting gene expression.

## 1. Introduction

Xerostomia is known as a subjective complaint of dry mouth and is caused by a reduction in salivary secretion and by changes in the composition of saliva related to the decline in the function of salivary glands [1,2,3]. There are several factors which lead to xerostomia, including systemic disease such as diabetic mellitus, autoimmune diseases, and renal failure [1], as well as the use of certain medications, such as antiallergy agents, antianxiety agents, anticholinergic agents, antidepressants, calcium channel blockers and anorexiants [4]. Biological aging also induces xerostomia [5,6,7,8,9,10]. Furthermore, xerostomia leads to infectious conditions, such as periodontal disease and caries dentium, in the oral cavity [11]. These oral infections aggravate diabetes [12] and increase the risk of atherosclerosis and cardiovascular disease [13]. Thus, xerostomia lies at the root of degenerative illness. 

Salivary glands are subdivided into three major glands (the parotid, submandibular and sublingual glands) and numerous minor glands. These glands are innervated by autonomic nerves [14]. The cholinergic parasympathetic nerve innervates all salivary glands and the parasympathetic nerve derived from the superior salivary nucleus innervates the submandibular glands (SMGs) and sublingual glands (SLGs), while parotid glands are innervated by the nerve originated from the inferior salivary nucleus. Minor salivary glands, in turn, are innervated by the parasympathetic fibers of the buccal branch of the mandibular nerve, whereas adrenergic sympathetic innervation of SMGs and parotid glands comes from the thoracic segment of spinal cord. The SLGs and minor salivary glands receive a sparse sympathetic nerve fiber. In parasympathetic and/or sympathetic nerves, non-adrenergic and non-cholinergic neurotransmitters, such as substance P, vasoactive intestinal peptide (VIP), neuropeptide Y, neurokinin A, and cholecystokinin (CCK), regulate nerve activity [15,16].

We previously reported that the administration of whey from the milk of Jersey cattle mitigates age-dependent atrophy and functional decline of salivary glands accompanied with changes in gene expression [17]. The major components of whey proteins are of β-lactoglobulin, α-lactalbumin, bovine serum albumin, and immunoglobulin, representing 50%, 20%, 10% and 10% of the whey fraction, respectively [18]. Although β-lactoglobulin A and B are the most prevalent variants, the β-lactoglobulin C variant is observed in Jersey cattle. There is one amino acid difference between the B variant (Gln^59^) and C variant (His^59^) [19,20]. In the present study, we investigated the effect of Leu^57^-Leu^58^-His^59^-Lys^60^ (LLHK), Leu^58^-His^59^-Lys^60^ (LHK) and His^59^-Lys^60^ (HK) peptides on the gene expression profile, age-dependent atrophy and dysfunction of rat salivary glands. 

## 2. Experimental Section

### 2.1. Experimental Animals and Collection of Saliva

Twenty-week-old male Wistar rats were purchased from SLC, Inc. (Shizuoka, Japan) and given standard laboratory chow (MF; Oriental Yeast, Tokyo, Japan) and water ad libitum. At the age of 80 weeks, rats were divided into the following four experimental groups (*n* = 3 per group): (I) water-treated control group, (II) LLHK-treated group, (III) LHK-treated group, and (IV) HK-treated group. They were provided water, LLHK (1 mg/mL) (Peptide Institute, Inc. Osaka, Japan), LHK (0.75 mg/mL) (Peptide Institute, Inc. Osaka, Japan), or HK (0.6 mg/mL) (Bachem AG, Bubendorf, Switzerland) ad libitum for 4 weeks. Five-week-old male Wistar rats were also purchased from SLC, Inc. and given a standard laboratory chow and water ad libitum until 16 weeks old. The animals were housed in an environmentally controlled room (22 ± 2 °C) with a 12-h light/dark cycle in accordance with the guidelines of Tokushima University Animal Experiment Committee. Whole saliva was collected from rats during the first 5–15 min after intraperitoneal administration of cevimeline hydrochloride (10.0 mg/kg) (Wako, Pure Chemical. Ind., Osaka, Japan) by a micropipette and placed in Eppendorf tubes kept on ice. Before and after the collection of saliva, the weight of each tube was measured. Salivary glands were rapidly harvested from rats euthanized by spinal dislocation under inhalation anesthesia. All animal procedures were approved by Tokushima University Animal Experiment Committee (TA14120 approved on 1 October 2014, T29-76 approved on 15 November 2017, and T29-124 approved on 1 April 2018) and the Ethics Committee of Animal Experiments in Suntory (APRV000644 approved on 21 May 2018).

### 2.2. Histological Analysis 

Salivary glands for light microscopy examination were fixed by immersion in 10% phosphate-buffered formalin and then processed for paraffin sectioning. Routinely, 5-µm sections were cut and stained with hematoxylin and eosin. 

### 2.3. Isolation of Total RNA 

The total RNA from SLGs of 3 control (water-treated 84-week-old) rats and 3 experimental (LHK-treated 84-week-old) rats was isolated using an RNeasy Mini Kit (Qiagen GmbH, Hilden, Germany) and QIAcube (Qiagen GmbH). RNA purity was quantitated by an absorbance ratio of 260/280 nm using a ND-1000 spectrophotometer (NanoDrop Technologies, Wilmington, DE, USA). The integrity of the RNA was assessed by Agilent 2100 Bioanalyzer (Agilent, Santa Clara, CA, USA). The total RNA samples which had a ratio more than 2.0 and an integrity greater than 9.0 were used in this study. 

### 2.4. DNA Microarray Experiments 

Cyanine-3 (Cy3)-labeled cRNA was generated from 0.15 µg total RNA by Low Input Quick Amp Labeling Kit, One-Color (Agilent) and then purified by RNeasy column (QIAGEN, Valencia, CA, USA). cRNA yield and labeling efficiency were validated by ND-1000 spectrophotometer. Then, 0.6 µg of Cy3-cRNA (more than 6 pmol Cy3/µg of cRNA) was fragmented for 30 min at 60 °C in a reaction mixture of 25 µL containing fragmentation buffer (Agilent) and blocking reagent (Agilent). After fragmentation, 25 µL of hybridization buffer was added to the sample and then hybridized to SurePrint GE Unrestricted Microarrays (Agilent, G2519F) for 17 h at 65°C in a rotating hybridization oven. The array was washed twice in GE Wash Buffer (Agilent) and immediately dried. After washing on the DNA Microarray Scanner (Agilent, G2505C), using one color scan setting for 8 × 60 k array slides, the slides were scanned (Scan Area: 61 × 21.6 mm, Scan resolution: 5 µm, dye channel: green, PMT: 100%). This array allowed us to analyze 30,584 Entrez known and unknown genes. 

### 2.5. Analysis of Microarray Data 

The scanned images were preprocessed by Feature Extraction Software 10.7.1.1 (Agilent) and then the data were imported into GeneSpring GX software version 14.9 (Agilent). After normalizing the array data, the restriction lists and functional classifications were summed in the comparison of data from 3 different experiments. The signal levels of the genes which were upregulated or downregulated by at least 2.0-fold were further analyzed. The expression change was taken as informative when the *p* value was <0.05. 

### 2.6. Measurement of α-Amylase Activity in Parotid Glands and Saliva

The α-amylase activity in parotid gland homogenate and saliva was measured as described by Bernfeld using amylose (Sigma Chemical Co., St. Louis, MO, USA) as the substrate [21]. The incubation mixture consisted of 1% soluble amylose in 0.02 M phosphate buffer (pH 6.9) containing 0.0067 M NaCl. In order to measure α-amylase activity, NaCl was added. After incubation at 20 °C for 3 min, the reaction was stopped by the addition of 1% dinitrosalicylic acid. The tube was heated for 5 min in boiling water. After cooling, absorbance was measured at 540 nm. Amylase activity was expressed as units per mg protein of parotid gland or ml of saliva, where 1 U amylase was defined as the quantity of enzyme that generated 1 mg of maltose in 1 min at 20 °C. 

### 2.7. Statistical Analysis

Data are expressed as the mean value ± standard error. To test for statistically significant differences between two groups, a paired Student’s *t* test was used for biochemical tests. In microarray analysis, a moderated *t* test was used. A *p* value of less than 0.05 was considered to be statistically significant.

## 3. Results

### 3.1. Effects of LLHK, LHK, and HK on Animal Body Weights, Salivary Glands Weights, and Salivary Volume 

In water-treated (control) older rats and peptide (LLHK, LHK, or HK)-treated older rats, the body weights at the beginning and end of the study were not significantly different (Table 1). In younger water-treated rats, there was a significant difference in body weight at the beginning and at the end of the study.

The weights of the parotid glands, SMGs, and SLGs and salivary volumes were significantly higher in LLHK- or LHK-treated older rats compared to water-treated older rats (Table 2). However, in the weights of parotid glands from HK-treated older rats, there was not a significant difference compared to those from water-treated older rats.

### 3.2. Effects of LLHK, LHK, and HK on the Morphology of Salivary Glands 

**Figure 1:**
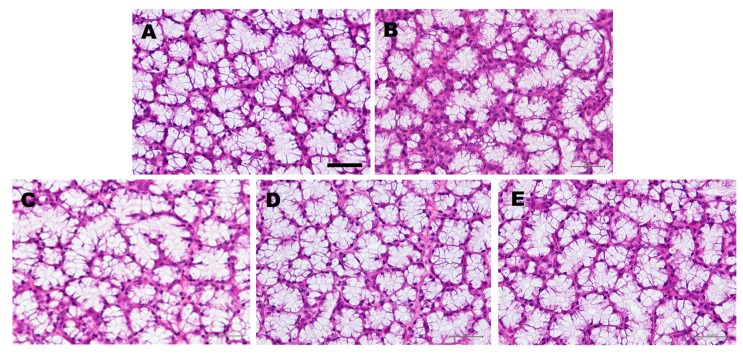
Effect of water-, Leu^57^-Leu^58^-His^59^-Lys^60^ (LLHK-), Leu^58^-His^59^-Lys^60^ (LHK)-, or His^59^-Lys^60^ (HK)-treatment on the morphology of sublingual glands (SLGs). The younger rats were given water (**A**). The older rats were given water- (**B**), LLHK- (**C**), LHK- (**D**), or HK- (**E**) for 1 month. Bar, 50 µm.

To evaluate the effect of the administration of LLHK, LHK, or HK on the morphology of the SLGs, we performed histological staining on SLGs tissue sections using hematoxylin and eosin (Figure 1). Acinar cell atrophy was detected in the SLGs of water-treated older rats (Figure 1B), but not in the SLGs of the younger water-treated rats (Figure 1A) or the SLGs of LLHK-, LHK-, or HK-treated older rats (Figure 1C–E). These results suggest that LLHK-, LHK-, or HK-treatment prevented the age-dependent atrophy of acinar cells of SLGs.

### 3.3. Effect of LHK on Gene Expression Profiles of Salivary Glands

Microarray is a predominant technology that enables the concurrent measurement of the expression levels of plethora genes. The resulting expression data lead to a tremendous understanding of cellular processes on the molecular level. Total RNA was isolated from the SLGs of three water-treated older rats and three LHK-treated older rats. 

Of 30,584 genes in Entrez, 39 known genes relevant to major salivary protein and salivary gland homeostasis were upregulated by at least 2.0-fold (*p ≤* 0.05) in the LHK-treated group compared to the water-treated group (Table 3 and Table S1). Other 39 known genes which have few relations with major salivary protein and salivary gland homeostasis, 12 ENSRNOT (rat-specific) genes, and 12 uncharacterized predicted genes were upregulated by at least 2.0-fold (*p ≤* 0.05) in the LHK-treated group compared to the water-treated group (Table S1). The upregulated known genes included those encoding major salivary families of specific secretory proteins [22], i.e., proline-rich proteins (*Prpmp5*, *Prb3*, *Prp2*, *Prb1*, *Prp15*), cystatin genes (*Cst5*, *Cyss*, *Vegp2*), and amylase genes (*Amy1a*, *Amy2a3*). The levels of transcription factor beta 2 (*Tfap2b*) and androgen-related protein (*Smr3a*) genes were also increased in the LHK-treated group compared to the water-treated group.

Of 30,584 genes in Entrez, 45 known genes relevant to salivary gland homeostasis were downregulated by at least 2.0-fold (*p* ≤ 0.05) in the LHK-treated group compared to the water-treated group (Table 4 and Table S2). Other 36 known genes which have few relations with salivary gland homeostasis, 37 ENSRNOT genes, and 49 uncharacterized predicted genes were downregulated by at least 2.0-fold (*p* ≤ 0.05) in the LHK-treated group compared to the water-treated group (Table S2). Among the downregulated genes, there were no major salivary families of specific secretory proteins. 

The transcription factor Activator Protein-2 (AP-2) gene (*Tcfap2b*), which is a salivary gland-specific transcription factor gene [23], was induced by LHK administration, suggesting that the tissue-specific transcription factor AP-2 participates in the expression of salivary gland-specific genes in LHK-treated rats. 

### 3.4. Effects of LLHK, LHK, and HK on Amylase Avtivity of Salivary Glands and Saliva 

To evaluate the effect of the administration of LLHK, LHK, or HK on the function of salivary glands, the amylase activity of parotid glands and saliva was measured as a marker of function (Table 5). After LLHK-, LHK-, or HK-treatment, the amylase activity in both the parotid glands and saliva increased significantly in comparison with water treatment. As shown in Table 2, salivary secretion was also increased by the administration of LLHK, LHK, or HK. Total amylase activity was calculated in whole saliva secreted during 5-15 min and expressed as U per whole saliva during 5–15 min in Table 5. The recovery of amylase activity with these peptides was remarkable. These results suggest that LLHK-, LHK-, or HK-treatment prevented the age-dependent malfunction of salivary glands.

## 4. Discussion

This is the first report showing that the administration of LLHK, LHK, and HK prevented and reversed age-dependent atrophy and functional decline of the salivary glands via changes in gene expression. LLHK, LHK, and HK are fragments of β-lactoglobulin C from Jersey cattle. Beta-lactoglobulin, a major whey protein, is an important source of biologically active peptides [24]. These peptides have been reported to play many crucial physiological roles and have anti-hypertensive, anti-oxidation, antimicrobial, anti-carcinogenic, pathogen adhesion, immunomodulating, and opioid activities, and they can decrease body cholesterol levels. Although many fragments from β-lactoglobulin have been characterized, there have been no investigations of LLHK, LHK, and HK to our best knowledge.

Saliva has important functions, such as in mastication, oral microbial defense, gustation, lubrication, speech, deglutition, digestion, mineralization of teeth, and the protection of mucosal tissues [25]. These essential functions of saliva are strictly connected to its volume and composition when secreted from salivary glands [26]. During aging, salivary volume decreases and the composition of saliva changes [2]. The administration of LLHK, LHK, and HK upregulated major salivary protein genes, such as proline-rich proteins (*Prpmp5*, *Prb3*, *Prp2, Prb1, Prp15*), cystatins (*Cst5*, *Cyss, Vegp2*), amylases (*Amy1a, Amy2a3*), and lysozyme (*Lyzl1*), suggesting that these peptides Change to “15”.reversed the age-dependent decline of salivary function.

Parotid glands and SMGs are innervated by cholinergic and adrenergic nerves. In addition to these nerves, the VIP and His-Met (HM) immunoreactive nerve, which is a non-adrenergic and non-cholinergic nerve, is in close proximity to the acini and ducts of parotid glands and SMGs [15]. HM is known to have the same precursor as VIP. It is possible that LLHK, LHK, and HK have an effect on non-adrenergic and non-cholinergic nerves in salivary glands. Immunocytochemical investigations have demonstrated the presence of a number of neuropeptides surrounding the inferior salivary nucleus [27]. Although it is uncertain that the HM immunoreactive nerve remains in existence in the inferior salivary nucleus, the oral administration of LLHK, LHK, and HK may influence central and/or peripheral non-adrenergic and non-cholinergic nerves.

Acinar cell atrophy, cytoplasmic vacuolization, and lymphocyte infiltration are detected in the SMGs of aged mice [8]. Meanwhile, in humans, the aging effects marked by a progressive decrease in glandular volume and acini volume accompanied by an increase in the volume of ducts, stroma, and adipose tissues are observed in SLGs [28]. The transcriptional changes by LHK-administration occurred within 1 week, but it took several weeks for the restoration of morphological decline (unshown data). In this study, we showed that the 1-month administration of LLHK, LHK, and HK peptides derived from β-lactoglobulin C prevented and/or reversed the age-dependent atrophy of SLGs.

Gly-His-Lys (GHK) is a peptide isolated from human serum [29] that has various biological functions, including in liver growth, angiogenesis, and nerve outgrowth, as well as anti-oxidation, anti-inflammatory, anti-cancer, anti-pain, and cell protective and regenerative actions [30]. GHK has also been shown to act on the expression of genes relevant to nervous system functioning and cognitive decline [31]. GHK has a high affinity for ionic copper, and studies have reported that, upon the formation of the GHK-Cu complex, these functions are advanced [32]. Furthermore, it has been reported that GHK changes the gene expression pattern in lungs via the activation of transforming growth factor beta (TGF-β) [33].

LHK treatment induced the expression of the *Tcfap2β* gene in salivary glands. As reported previously, whey consumption induces *Tcfap2β* expression [17]. The AP-2 family of transcription factors consists of five members, *AP-2α, 2β, 2γ, 2δ*, and *2ε*, each encoded by a separate gene [34], and is known to have tissue-specific expression to regulate the expression of other genes [23]. The transcription factor AP-2 promotes cartilage and skeletal development and reduces apoptotic cell death in renal epithelial cells [35]. LLHK, LHK, and HK, which are derived from β-lactoglobulin C, may induce salivary gland-specific genes via the transcription factor AP-2β.

In this study, we investigated the effects of LLHK, LHK, and HK on salivary glands. As the structure of LHK and GHK are very similar, LLHK, LHK, and HK may also have multiple functions.

## 5. Conclusions

Long-term oral administration of LLHK, LHK, and HK regulated gene expression and led to the prevention and/or reversal of age-dependent atrophy and functional decline of salivary glands.

## Figures and Tables

**Table 1:** Body weights in water-treated and peptide-treated groups.

	Group	Initial	Final
Body weight (g)	Older/Water	446 ± 12	440 ± 12
	Older/LLHK	444 ± 21	445 ± 23
	Older/LHK	457 ± 17	458 ± 28
	Older/HK	448± 7	429± 6
	Younger/Water	301 ± 11	336 ± 13 *

Data represent means ± SE (*n* = 3); * *p* < 0.05 compared to water-treated older rat.

**Table 2:** Salivary gland weight and salivary volume in water-treated and peptide-treated groups.

	Group	Older	Older	Older	Older	Younger
Weight		Water	LLHK	LHK	HK	Water
Salivary glands (mg)	Parotid gland	178 ± 12	244 ± 23 **	249 ± 19 **	191± 8	196± 2 *
	Submandibular gland	268± 5	306 ± 18 *	298 ± 28 *	285 ± 22 *	263 ± 23
	Sublingual	45.8 ± 5.9	65.9 ± 2.5 *	53.1 ± 2.4 *	51.5 ± 3.5 *	48.4 ± 4.5
Salivary volume (mg/5–15min)		132 ± 18	254 ± 26 **	360 ± 12 **	399± 29 **	188 ± 1 5 *

Data represent means ± SE (*n* = 3–6); * *p* < 0.05; ** *p* < 0.01 compared to water-treated older rat.

**Table 3:** Genes upregulated by >2.0-fold (*p* < 0.05) in SLGs from LHK-treated group compared to those from water-treated group.

Gene Name	Gene Symbol	FC	Accession No.	*p*-Value
**Major Salivary Protein Gene**				
Proline-Rich Protein				
proline-rich protein MP5	Prpmp5	1664.69	NM_172065	3.80 × 10^−4^
proline-rich protein BstNI subfamily 3	Prb3	1034.47	NM_139184	0.002
proline-rich protein 2	Prp2	29.23	NM_001013211	0.041
proline-rich protein BstNI subfamily 1	Prb1	8.33	NM_172064	0.046
proline-rich protein 15	Prp15	2.07	NM_012632	0.047
Cystatin Proteinase Inhibitor				
cystatin D	Cst5	325.83	NM_001108961	5.23 × 10^−4^
von Ebners gland protein 2	Vegp2	4.38	NM_053574	0.007
cystatin S	Cyss	2.88	NM_198685	0.048
Amylase				
amylase, alpha 1A	Amy1a	27.81	NM_001010970	0.045
amylase 2a3	Amy2a3	23.97	NM_031502	0.047
Secretion-Related Protein				
cysteine-rich secretory protein 3	Crisp3	561.42	NM_022859	1.05 × 10^−4^
solute carrier family 45, member 1	Slc45a1	3.128	NM_144747	3.60 × 10^−4^
synaptotagmin 17	Syt17	2.79	NM_138849	0.019
calcium binding protein 1	Cabp1	2.49	NM_001033676	0.035
solute carrier family 4 member 10	Slc4a10	2.38	NM_178092	0.036
lysozyme-like 1	Lyzl1	2.36	NM_001108882	0.038
calcium dependent secretion activator	Cadps	2.25	NM_013219	0.050
solute carrier family 10 member 2	Slc10a2	2.21	NM_017222	0.010
**Salivary Gland Homeostasis Genes**				
Androgen Related Protein				
submaxillary gland androgen regulated protein 3A	Smr3a	277.48	NM_001017497	0.004
variable coding sequence A2	Vcsa2	4.60	NM_198729	0.046
androgen-dependent TFPI-regulating protein	Adtrp	3.22	NM_001014144	0.046
Signal Transduction				
tachykinin, precursor 1	Tac1	7.27	NM_012666	0.034
Ras-like without CAAX 2	Rit2	3.44	NM_001013060	7.03 × 10^−4^
serine/threonine kinase 32C	Stk32c	2.61	NM_001108922	0.001
acetylcholinesterase	Ache	2.47	NM_172009]	0.020
taste receptor, type 2, member 117	Tas2r117	2.44	NM_001166682	0.049
protein tyrosine phosphatase, non-receptor type 22	Ptpn22	2.17	NM_001106460	0.026
G protein subunit gamma 2	Gng2	2.05	NM_031754	0.043
Transcriptional Regulator				
deoxyribonuclease 1	Dnase1	1361.76	NM_013097	3.04 × 10^−4^
transcription factor AP2-beta	Tfap2b	6.88	NM_001106896	4.90 × 10^−2^
zinc finger protein 157	Zfp157	3.34	NM_001170404	0.022
ganglioside-induced differentiation-associated protein 1	Gdap1l1	2.42	NM_001107798	0.008
Growth				
fibroblast growth factor receptor 4	Fgfr4	4.14	NM_001109904	0.034
insulin-like growth factor binding protein	Igfals	2.19	NM_053329p	0.015

(*n* = 3).

**Table 4:** Genes down-regulated by >2.0-fold (*p* < 0.05) in SLGs from LHK-treated group compared to those from water-treated group.

Gene Name	Gene Symbol	FC	Accession No.	*p*-Value
Salivary Gland Homeostasis Protein Genes				
Signal Transduction				
Wnt family member 9B	Wnt9b	−2.72	NM_001107055	0.042
adenylate kinase 7	Ak7	−2.20	NM_001108055	0.047
serpin family B member 3	Serpinb3	−2.15	NM_001008887	0.031
cyclin-dependent kinase inhibitor 1A	Cdkn1a	−2.04	NM_080782	0.016
hormonally upregulated Neu-associated kinase	Hunk	−2.02	NM_001191662	0.024
Transcriptional Regulator				
actin-binding Rho activating protein	Abra	−3.32	NM_175844	0.003
early growth response 2	Egr2	−3.21	NM_053633	0.015
D-box binding PAR bZIP transcription factor	Dbp	−2.56	NM_012543	0.040
basic helix-loop-helix family, member e22	Bhlhe22	−2.32	NM_001108940	0.003
activating transcription factor 3	Atf3	−2.39	NM_012912	0.017
PARN like, ribonuclease domain containing 1	Pnldc1	−2.26	NM_001025724	0.014
homeo box A2	Hoxa2	−2.19	NM_012581	0.041
nuclear receptor subfamily 4, group A, member 2	Nr4a2	−2.16	NM_019328	0.016
TGFB-induced factor homeobox 2	Tgif2	−2.13	NM_001134983	0.035
Metabolism				
N-deacetylase and N-sulfotransferase 4	Ndst4	−9.65	NM_001191849	0.048
fatty acyl CoA reductase 1	Far1	−2.84	NM_001011933	0.026
solute carrier family 27 member 5	Slc27a5	−2.23	NM_024143	0.002
ring finger protein 222	Rnf222	−2.28	NM_001108828	0.046
ADAM metallopeptidase with thrombospondin type 1 motif, 18	Adamts18	−2.10	NM_001191944	0.023
tryptophan hydroxylase 2	Tph2	−2.09	NM_173839	0.031

(*n* = 3).

**Table 5:** Amylase activity in parotid gland and saliva.

	Salivary Gland (U/mg Protein)	Saliva (U/mL)	Saliva (U/Whole Saliva during 5–15 min)
Older/Water	148± 4	9.17 ± 0.70	1.21 ± 0.09
Older/LLHK	201 ± 16 *	21.40 ± 3.59 **	5.44 ± 0.91 **
Older/LHK	246 ± 17 **	21.66 ± 2.29 **	7.80 ± 0.82 **
Older/HK	216 ± 20 **	16.20 ± 2.70 **	6.46 ± 1.08 **
Younger/Water	244 ± 8 **	25.84 ± 2.24 **	4.86 ± 0.42 **

Data represent means ± SE (*n* = 3–6); * *p* < 0.05; ** *p* < 0.01 compared to water-treated older rat.

## Supplementary Materials

The following are available online at https://www.mdpi.com/2227-9059/8/2/38/s1.

## Abbreviations

LLHK	Leu-Leu-His-Lys, Leucyl-Leucyl-Histidyl-Lysine
LHK	Leu-His-Lys, Leucyl-Histidyl-Lysine
HK	His-Lys, Histidyl-Lysine
SMGs	Submandibular glands
SLGs	Sublingual glands
VIP	Vasoactive intestinal peptide
CCK	Cholecystokinin
FC	Fold change
HM	His-Met, Histidyl-Metionine
TGF-b	Transforming growth factor-beta
AP-2b	Activator Protein-2 beta
