# Supplementary material for: Long-Term Oral Administration of LLHK, LHK, and HK Alters Gene Expression Profile and Restores Age-Dependent Atrophy and Dysfunction of Rat Salivary Glands
Source: Biomedicines. 2020 Feb 20;8(2):38. doi: 10.3390/biomedicines8020038 (PMC7168239; doi:10.3390/biomedicines8020038)
Supplement: Supplementary file 1 [file biomedicines-08-00038-s001.pdf]

Table S1. Genes up-regulated by >2.0-fold (p<0.05) in SLGs from LHK-treated group compared to those from water-treated group.

GeneName	GeneSymbol	FC	Accession no	p-value
**Salivary gland homeostasis genes**				
**Cytoplasm components**				
tubulin, beta 3 class III	Tubb3	3.73	NM_139254	0.040
synapsin I	Syn1	2.22	NM_019133	0.045
tubulin, alpha 3A	Tuba3a	2.19	NM_001040008	0.046
lipocalin 6	Lcn6	2.14	NM_001001519	0.034
**Other genes**				
RGD1559532	RGD1559532	75.76	NM_001024983	0.034
apelin receptor	Aplnr	67.78	NM_031349	0.034
BPI fold containing family B, 1	Bpifb1	32.58	NM_001077680	0.014
BPI fold containing family A, 2	Bpifa2	5.38	NM_052808	0.026
enamelin	Enam	4.63	NM_001106001	0.048
ELAV like RNA binding protein 4	Elavl4	3.32	NM_001077651	0.046
fibroblast growth factor 4	Fgf4	3.23	NM_053809	0.014
granzyme C	Gzmc	3.01	NM_134332	0.031
olfactory receptor 1519	Olr1519	2.97	NM_001000037	0.032
interleukin 21	Il21	2.82	NM_001108943	0.002
granzyme A	Gzma	2.73	NM_153468	0.041
paraneoplastic Ma antigen family, 5	Pnma5	2.69	NM_001107579	0.018
thioesterase superfamily member 5	Them5	2.60	NM_001108558	0.013
sterile alpha motif domain containing 3	Samd3	2.58	NM_001172116	0.003
gigaxonin	Gan	2.49	NM_001107434	0.026
olfactory receptor 1598	Olr1598	2.47	NM_001000910	0.004
transgelin 3	Tagln3	2.44	NM_031676	0.005
amine oxidase, copper containing 2	Aoc2-ps1	2.43	NR_033180	0.046
killer cell lectin-like receptor family I ,2	Klri2	2.37	NM_001012648	0.011
cytokine receptor-like factor 3	Crlf3	2.37	NM_001168612	0.034
olfactory receptor 921	Olr921	2.25	NM_001001385	0.017
ATPase H+/K+ transporting beta subunit	Atp4b	2.24	NM_012510	0.008
V-set pre-B cell surrogate light chain 3	Vpreb3	2.24	NM_001108930	0.042
essential meiotic structure endonuclease 1	Eme1	2.22	NM_001105830	0.018
pyruvate kinase, muscle	Pkm	2.19	NM_053297	0.003
major facilitator superfamily domain 6-like	Mfsd6l	2.18	NM_001105787	0.049

olfactory receptor 515	Olr515	2.14	NM_001000315	0.007
Ly6/neurotoxin 1	Lynx1	2.14	NM_001130546	0.043
amelogenin, X-linked	Amelx	2.13	NM_001271074	0.001
calsyntenin 2	Clstn2	2.12	NM_134377	0.030
translocase of inner mitochondrial 44	Timm44	2.09	NM_017267	0.005
sterile alpha and TIR motif containing 1	Sarm1	2.09	NM_001105817	0.026
nuclear factor of activated T-cells 2	Nfatc2	2.09	XM_017591801	0.025
myosin binding protein C, slow type	Mybpc1	2.08	NM_001100758	0.002
hes-related family bHLH transcription factor with YRPW motif-like	Heyl	2.04	NM_001107977	0.029
T cell activation inhibitor, mitochondrial	Tcaim	2.04	NM_001110838	0.005
histidine decarboxylase	Hdc	2.00	NM_017016	0.036
**ENSRNOT genes**				
family with sequence similarity 13, C	Fam13c	4.59	ENSRNOT00000051491	80.04
methyltransferase like 8		3.86	ENSRNOT00000075860	0.020
family with sequence similarity 160, B1	Fam160b1	2.38	ENSRNOT00000023247	0.047
similar to KIAA1549 protein	RGD1306271	2.37	ENSRNOT00000040391	0.049
pappalysin 2	Pappa2	2.34	ENSRNOT00000089332	0.030
IgLON family member 5	Iglon5	2.28	ENSRNOT00000024150	0.026
ubiquitin specific peptidase 27,		2.28	ENSRNOT00000003800	0.020
ly-6/neurotoxin-like protein 1-like		2.26	ENSRNOT00000073179	0.024
solute carrier family 18 member B1	Slc18b1	2.24	ENSRNOT00000021944	0.035
RAD51 associated protein 2		2.14	ENSRNOT00000075503	0.004
nuclear receptor subfamily 1, group H, 5	Nr1h5	2.01	ENSRNOT00000036229	0.009
zinc finger protein 182-like	LOC102555377	2.09	ENSRNOT00000034746	0.047
**Predicted genes**				
uncharacterized	LOC102555043	2.96	PREDICTED:XR_001840966	0.026
uncharacterized	LOC102553665	2.96	PREDICTED:XR_344513	0.024
uncharacterized transcript variant X4	LOC103692336	2.91	PREDICTED:XR_001843643	0.024
uncharacterized	LOC102556336	2.87	PREDICTED:XR_597473	0.001
similar to RIKEN cDNA 5031410I06, transcript variant X9	LOC679711	2.83	PREDICTED:XM_017590496	0.041
uncharacterized	LOC102554987	2.54	PREDICTED:XR_590492	0.004
cDNA clone urrg1-00021-g10 5'		2.48	AMGNNUC:CB793397	0.040
uncharacterized, transcript variant X1	LOC103692702	2.48	PREDICTED:XR_593187	0.036
cDNA clone LIONp463H04130 3'		2.45	CR475266	0.003

uncharacterized, transcript variant X1	LOC102549525	2.40	PREDICTED:XR_592148	0.023

(n=3)

Table S2. Genes down-regulated by >2.0-fold (*p*<0.05) in SLGs from LHK-treated group compared to those from water-treated group.

GeneName	GeneSymbol	FC	Accession no	p-value
**Salivary gland homeostasis protein genes**				
**Membrane components**				
glycoprotein 2	Gp2	-8.45	NM_134418	0.003
transmembrane protein 67	Tmem67	-8.07	NM_001107916	0.021
solute carrier family 22, member 20	Slc22a20	-3.02	NM_001106327	0.028
protocadherin gamma subfamily A, 5	Pcdhga5	-2.69	NM_001037137	0.025
Eph receptor B2	Ephb2	-4.80	NM_001127319	0.004
chloride channel, voltage-sensitive 2	Clcn2	-2.59	NM_017137	0.032
calpain 3	Capn3	-2.63	NM_017117	0.016
oxidized low density lipoprotein receptor 1	Olr1	-2.49	NM_133306	0.044
glycoprotein IX (platelet)	Gp9	-2.48	NM_001031825	0.005
transmembrane protein 170B	Tmem170b	-2.08	NM_001008774	0.007
**Cytoplasm components**				
zinc finger protein 13	Zfp13	-5.82	NM_001105765	0.018
kelch repeat and BTB domain containing 4	Kbtbd4	-4.39	NM_001107746	3.48E-04
PICALM interacting mitotic regulator	Pimreg	-2.75	NM_001113781	0.037
profilin 3	Pfn3	-2.23	NM_001109487	0.011
keratin 24	Krt24	-2.58	NM_001004131	0.017
DnaJ heat shock protein family (Hsp40) B13	Dnajb13	-2.11	NM_001005885	0.028
family with sequence similarity 71, member B	Fam71b	-2.10	NM_001025031	0.026
FERM domain containing 3	Frmd3	-2.10	NM_001106662	0.048
**Immune response**				
T-cell receptor beta chain	Tcrb	-5.07	CO560445	0.005
defensin RatNP-3 precursor, mRNA sequence	RatNP-3b	-2.79	BQ200749	0.018
complement C9	C9	-2.36	NM_057146	0.035
autoimmune regulator	Aire	-2.31	NM_001106379	0.003
tumor necrosis factor	Tnf	-2.31	NM_012675	0.012
interleukin 1 beta	Il1b	-2.09	NM_031512	0.002
**Other genes**				

olfactory receptor 673	Olr673	-9.36	NM_001000351	0.049
glutamate ionotropic receptor kainate type subunit 2	Grik2	-5.42	NM_019309	0.013
olfactory receptor 1525	Olr1525	-5.24	NM_001000529	3.99E-04
potassium channel tetramerization domain 4	Kctd4	-4.15	NM_001109650	0.010
olfactory receptor 1122	Olr1122	-3.95	NM_001000882	0.005
olfactory receptor 371	Olr371	-3.76	NM_001000258	0.048
HORMA domain containing 1	Hormad1	-3.26	NM_001108949	0.009
notochord homeobox	Noto	-3.17	NM_001192014	0.022
olfactory receptor 219	Olr219	-2.98	NM_001001372	0.041
RT1 class I, M1, gene 5	RT1-M1-5	-2.90	NM_001168332	0.047
meiotic double-stranded break formation protein 4	Mei4	-2.84	NM_001109378	0.010
MOS proto-oncogene, serine/threonine kinase	Mos	-2.83	NM_020102	0.005
olfactory receptor 1149	Olr1149	-2.75	NM_001000874	0.022
similar to RIKEN cDNA 1700063I17	RGD1560958	-2.66	NM_001109157	0.021
Myb/SANT DNA binding domain containing 1	Msantd1	-2.59	NM_001109089	0.042
crystallin, beta A1	Cryba1	-2.58	NM_013056	0.007
olfactory receptor gene Olr1686	Olr1686	-2.53	NM_001001373	0.035
C-type lectin domain family 3, member A	Clec3a	-2.56	NM_001108899	0.001
tubby-like protein 2	Tulp2	-2.37	NM_001012168	0.044
angiopoietin 2	Angpt2	-2.35	NM_134454	0.044
complexin 4	Cplx4	-2.32	NM_001191835]	0.034
sperm motility kinase 2A	Smok2a	-2.28	NM_001191619	0.047
olfactory receptor 1687	Olr1687	-2.27	NM_001000508	0.027
GIMAP family P-loop NTPase domain containing 1	Gimd1	-2.21	NM_001113782	0.006
olfactory receptor 60	Olr60	-2.19	NM_001000748	0.046
dendrin	Ddn	-2.17	NM_030993	0.041
olfactory receptor 1699	Olr1699	-2.15	NM_001001112	0.040
glutamate metabotropic receptor 2	Grm2	-2.15	NM_001105711	0.002
immediate early response 3 interacting protein 1	Ier3ip1	-2.12	NM_001047956	0.040
tektin 3	Tekt3	-2.12	NM_001024739	0.039
neutrophil cytosolic factor 2	Ncf2	-2.10	NM_001100984	0.032
Cd300 molecule-like family member F	Cd300lf	-2.10	NM_001025111	0.032
contactin associated protein-like 5A	Cntnap5a	-2.09	NM_001047865	0.038
ADAM metallopeptidase with thrombospondin type 1	Adamts1	-2.06	TC573828	0.038
hypothetical protein LOC689065	LOC689065	-2.06	NM_001109521	0.040
similar to immunoglobulin heavy chain		-2.04	XM_345749	0.029

ENSRNOT genes

MAP/microtubule affinity-regulating kinase like 1		-9.48	ENSRNOT00000058760	0.047
DAZ interacting protein 1, pseudogene 1		-7.26	ENSRNOT00000072327	0.040
leucine rich repeat containing 30	Lrrc30	-5.24	ENSRNOT00000049720	6.14E-04
ryanodine receptor 1		-5.05	ENSRNOT00000027893	0.031
WBP2 N-terminal like	Wbp2nl	-4.36	ENSRNOT00000010606	0.044
ATPase phospholipid transporting 8A1	Atp8a1	-4.03	ENSRNOT00000044017	0.029
Cd300 molecule-like family member D		-3.58	ENSRNOT00000077882	0.031
early growth response 1	Egr1	-2.86	ENSRNOT00000026303	0.050
unc-51 like kinase 4	Ulk4	-2.73	ENSRNOT00000074423	0.038
transmembrane protease, serine 11e		-2.69	ENSRNOT00000036948	0.033
LRRGT00141	LOC499235	-2.69	ENSRNOT00000045415	0.045
signal-regulatory protein alpha-like		-2.69	ENSRNOT00000071638	0.007
X-ray radiation resistance associated 1	Xrra1	-2.62	ENSRNOT00000087818	0.021
zinc finger and SCAN domain containing 4F	Zscan4f	-2.53	ENSRNOT00000030164	0.034
trichohyalin	Tchh	-2.52	ENSRNOT00000090212	0.004
neuroligin 1	Nlgn1	-2.51	ENSRNOT00000092660	0.008
SMG6 nonsense mediated mRNA decay factor	Smg6	-2.47	ENSRNOT00000052412	0.027
synaptonemal complex central element protein 1	Syce1l	-2.45	ENSRNOT00000045250	0.038
LRRGT00094		-2.40	ENSRNOT00000072833	0.034
disks large homolog 5-like		-2.33	ENSRNOT00000046644	0.006
acyl-protein thioesterase 1 pseudogen		-2.32	ENSRNOT00000011092	0.048
serine protease inhibitor, Kunitz type 4		-2.25	ENSRNOT00000019849	0.046
protocadherin beta 7	Pcdhb7	-2.23	ENSRNOT00000073425	0.029
ankyrin repeat domain 63	Ankrd63	-2.22	ENSRNOT00000056450	0.036
immunoglobulin superfamily, member 9B	Igsf9b	-2.20	ENSRNOT00000012391	0.005
multiple EGF-like-domains 11	Megf11	-2.18	ENSRNOT00000037941	0.043
similar to KIAA0825 protein	RGD1560883	-2.15	ENSRNOT00000071535	0.003
keratin associated protein 3-1	Krtap3-1	-2.14	ENSRNOT00000016752	9.019
collagen type XI alpha 2 chain	Col11a2	-2.14	ENSRNOT00000084117	0.041
cilia and flagella associated protein 70	Cfap70	-2.13	ENSRNOT00000035345	0.034
serine (or cysteine) peptidase inhibitor, clade B (ovalbumin), member 3A	Serpinb3a	-2.08	ENSRNOT00000003475	0.002
family with sequence similarity 47, A	Fam47a	-2.06	ENSRNOT00000005141	0.026
transmembrane protein 253	Tmem253	-2.06	ENSRNOT00000060521	0.044
EF-hand calcium binding domain 5	Efcab5	-2.03	PREDICTED:XM_006220732	0.036

hypothetical protein LOC689042		-2.02	ENSRNOT00000034929	0.017
ATP binding cassette subfamily B member 5	Abcb5	-2.02	ENSRNOT00000074197	0.036
**Predicted genes**				
sperm motility kinase 3-like	LOC103695049	-23.95	PREDICTED:XM_008774726	0.047
filaggrin family member 2	Flg2	-13.91	PREDICTED:XM_017596329	0.045
EF hand calcium binding domain 1	Efcab1	-6.42	PREDICTED:XM_017597950	0.019
olfactory receptor 143-like	LOC102554388	-6.23	PREDICTED:XM_008766089	0.044
similar to ankyrin repeat domain 26	LOC691693	-5.55	PREDICTED:XM_001079298	0.042
retinitis pigmentosa GTPase regulator interacting protein 1	Rpgrip1	-4.33	PREDICTED:XM_017599867	0.029
probable ATP-dependent RNA helicase DDX60-like	LOC100911190	-3.89	PREDICTED:XM_017600408	0.003
collagen alpha-1(XXIII) chain	LOC103693323	-3.84	PREDICTED:XM_017597627	0.031
disks large homolog 5-like	LOC102554774	-3.75	PREDICTED:XM_006251741	0.023
zinc finger protein 2 homolog	LOC108348729	-3.55	PREDICTED:XM_017587800	0.042
myosin heavy chain 13	Myh13	-3.54	PREDICTED:XM_008775759	0.046
regulatory factor X4	Rfx4	-3.44	PREDICTED:XM_006241183	0.039
heparan sulfate 6-O-sulfotransferase 2	Hs6st2	-3.31	PREDICTED:XM_017601970	0.010
coiled-coil domain containing 71-like	Ccdc71l	-3.14	PREDICTED:XM_003750141	0.014
zinc finger protein 945	Zfp945	-3.13	PREDICTED:XM_006228099	0.024
centromere protein O	Cenpo	-3.05	PREDICTED:XM_008764544	0.035
BTB domain containing 7	Btbd7	-2.95	PREDICTED:XM_008764820	0.038
NOP2/Sun RNA methyltransferase family member 7	Nsun7	-2.80	PREDICTED:XM_006250947	0.045
period circadian clock 1	Per1	-2.70	PREDICTED:XM_006246614	0.007
rCG23154-like	LOC680725	-2.67	PREDICTED:XR_146481	0.010
cytochrome P450, family 2, subfamily r, polypeptide 1	Cyp2r1	-2.64	PREDICTED:XM_008759769	0.039
E4F transcription factor 1	E4f1	-2.60	PREDICTED:XM_006246026	0.007
signal-regulatory protein alpha-like	LOC100365868	-2.59	PREDICTED:XM_008758154	0.002
BRCA2-interacting protein-like	LOC100360296	-2.59	PREDICTED:XM_003754681	0.050
similar to Discs, large homolog 5	RGD1563738	-2.55	PREDICTED:XM_008766093	0.039
exocyst complex component 2-like	LOC103689971	-2.51	PREDICTED:XM_006253876	0.027
rCG25329-like	LOC100360613	-2.44	PREDICTED:XR_001839588	0.049
transmembrane protein 132C	Tmem132c	-2.39	PREDICTED:XM_002724836	0.023
similar to RIKEN cDNA 9430015G10	RGD1311517	-2.34	PREDICTED:XM_008764363	0.002
proline-rich extensin-like protein	LOC103692530	-2.32	PREDICTED:XM_008776184	0.021
G protein-coupled receptor 152	Gpr152	-2.30	PREDICTED:XM_001068698	0.031
contactin associated protein-like 2	Cntnap2	-2.28	PREDICTED:XM_008762930	0.029
calcium binding and coiled-coil domain 2	Calcoco2	-2.25	PREDICTED:XM_008768252]	0.040

transient receptor potential cation channel, subfamily M, member 6	Trpm6	-2.24	PREDICTED:XM_006223673	0.005
similar to Spindlin-like protein 2	LOC367746	-2.23	PREDICTED:XM_008758379	0.016
partitioning defective 6 homolog gamma-like	LOC100909590	-2.20	PREDICTED:XM_003751813	0.040
autophagy related 10	Atg10	-2.19	PREDICTED:XM_006231757	0.037
similar to glyceraldehyde-3-phosphate dehydrogenase	RGD1560797	-2.16	PREDICTED:XR_340823	0.020
katanin p60 ATPase-containing subunit A-like 1-like	LOC100910196	-2.15	PREDICTED:XM_006248828	0.021
PX domain containing serine/threonine kinase	Pxk	-2.14	PREDICTED:XM_017599724	0.007
transmembrane protein 92-like	LOC103693451	-2.09	PREDICTED:XM_008768247	0.046
elastin microfibril interfacer 3	Emilin3	-2.07	PREDICTED:XM_006235495	0.044
H2A histone family, member Y	H2afy	-2.06	PREDICTED:XM_006253577	0.049
PCF11 cleavage and polyadenylation factor subunit	Pcf11	-2.06	PREDICTED:XM_008774627	0.043
glycine decarboxylase	Gldc	-2.05	PREDICTED:XM_006231210	0.030
proline rich 23 domain containing 2	Prr23d2	-2.05	PREDICTED:XM_008766601	0.003
ADMA metallopeptidase with thrombospondin 1 motif, 6	Adamts6	-2.01	PREDICTED:XM_017593685	0.027
carboxypeptidase O	Cpo	-2.01	PREDICTED:XM_017596898	0.026

(n=3)
